# Perioperative and oncologic outcomes of minimally-invasive surgery for renal cell carcinoma with venous tumor thrombus: a systematic review and meta-analysis of comparative trials

**DOI:** 10.1097/JS9.0000000000000405

**Published:** 2023-07-31

**Authors:** Kun-peng Li, Si-yu Chen, Chen-yang Wang, Xiao-ran Li, Li Yang

**Affiliations:** Department of Urology, The Second Hospital of Lanzhou University, Lanzhou, People’s Republic of China

**Keywords:** meta-analysis, minimally-invasive surgical procedures, open surgical procedures, renal cell carcinoma, venous tumor thrombus

## Abstract

**Background:**

The present study aimed to conduct a pooled analysis to compare the perioperative and oncologic outcomes of minimally-invasive radical nephrectomy with tumor thrombus (MI-RNTT) with open radical nephrectomy with tumor thrombus (O-RNTT).

**Methods:**

This study followed the Preferred Reporting Items for Systematic Reviews and Meta-Analyses (PRISMA) statement. Four electronic databases (PubMed, Embase, Web of Science, and the Cochrane Library database) were systematically searched to identify relevant studies published in English up to December 2022. The primary outcomes were perioperative results, complications, and oncologic outcomes. Review Manager 5.4 was used for this analysis.

**Results:**

In total, eight retrospective trials with a total of 563 patients were included. Compared to O-RNTT, MI-RNTT had shorter hospitalization time [weighted mean difference (WMD) -3.58 days, 95% CI: −4.56 to −2.59; *P*<0.00001), lower volumes of blood loss (WMD -663.32 ml, 95% CI: −822.22 to −504.42; *P*<0.00001), fewer transfusion rates (OR 0.18, 95% CI: 0.09–0.35; *P*<0.00001), fewer overall complications (OR 0.33, 95% CI: 0.22–0.49; *P*<0.00001), and fewer major complications s (OR 0.49, 95% CI: 0.24–1.00; *P*=0.05). However, operative time, intraoperative complications, mortality rate (intraoperative, within 30 days, and total mortality), overall survival, recurrence-free survival, and cancer-specific survival did not significantly differ between the two groups.

**Conclusions:**

MI-RNTT possesses more benefits than O-RNTT in terms of length of hospital stay, blood loss, and complications and provides comparable mortality rates and oncologic outcomes. However, more comprehensive and rigorous research is warranted to further validate the outcomes, which should include a larger sample size and comprehensive data from high-volume medical centers.

## Introduction

Highlights

- Most of the studies were published between 2019 and 2022.
- Minimally-invasive radical nephrectomy with tumor thrombus (MI-RNTT) has more advantages than open radical nephrectomy with tumor thrombus in perioperative outcomes.
- MI-RNTT has demonstrated comparable mortality rates and oncological outcomes to open radical nephrectomy with tumor thrombus.
- MI-RNTT could be regarded as a superior treatment option for patients diagnosed with renal cell carcinoma with venous tumor thrombus.

The incidence of inferior vena cava tumor thrombus in locally advanced renal cell carcinoma has been reported to be up to 10%^1^. Although surgical intervention is challenging, radical nephrectomy, and caval thrombectomy are the only recommended options in this setting. Indeed, radical nephrectomy and caval thrombectomy can effectively and safely improve the prognosis of patients with local renal cell carcinoma^2^. Despite advancements in minimally-invasive surgical procedures over the past few years, European guidelines still recommend open surgery as the gold standard for renal cell carcinoma with venous tumor thrombus^3^. However, open surgery has been found to be associated with a higher degree of trauma than minimally-invasive surgery^4^, as well as a significantly higher risk of perioperative mortality (4.6%), which are serious disadvantages of open surgery^5^.

Desai *et al*.^6^ presented their initial experience of laparoscopic radical nephrectomy with tumor thrombus (L-RNTT) in 2003. Furthermore, Abaza^7^ initially described a series of robotic radical nephrectomy with tumor thrombus (RA-RNTT) in 2011. Several studies have subsequently reported the feasibility of minimally-invasive radical nephrectomy with tumor thrombus (MI-RNTT) and demonstrated its safety and efficacy in treating renal cell carcinoma with venous tumor thrombus^8–10^. However, the operation space of laparoscopic and robotic surgeries is relatively confined and therefore necessitates the surgeon to be proficient in endoscopic vascular suturing, thereby increasing the complexity of the surgery^11^. Recently, some reports have attempted to compare the perioperative and oncologic outcomes of MI-RNTT and open radical nephrectomy with tumor thrombus (O-RNTT), but the results are controversial and have not been sufficiently comprehensive^12^. Moreover, studies with small sample sizes from a single-center prevented the acquisition of reliable outcomes.

Therefore, we conducted a meta‐analysis to evaluate the safety and efficacy of MI-RNTT versus those of O-RNTT for the treatment of renal cell carcinoma with venous tumor thrombus in an attempt to provide sufficient evidence to support clinical decisions.

## Methods

This meta-analysis was performed in accordance with the Preferred Reporting Items for Systematic Reviews and Meta-Analyses (PRISMA) statement 2020^13,14^ (Supplemental Digital Content 1, http://links.lww.com/JS9/A788). Quality assessment was performed according to Assessing the Methodological Quality of Systematic Reviews (AMSTAR) 2^15^ (Supplemental Digital Content 2, http://links.lww.com/JS9/A789) and was registered in PROSPERO.

### Literature search strategy, study selection, and data collection

A systematic search was performed to identify fully-published studies till December 2022 in the Science, PubMed, Web of Science, and the Cochrane Library databases. The search terms were as follows: (Robotic radical nephrectomy OR Robot-assisted radical nephrectomy OR Robot-assisted radical surgery) AND (Laparoscopic radical nephrectomy OR Laparoscopic radical surgery) AND (Kidney cancer OR Renal carcinoma OR Renal tumor OR Renal mass) AND (Inferior vena cava OR Thrombosis OR Thrombus OR Thrombectomy). Furthermore, the relevant references and abstracts were manually examined to avoid omissions and to expand the search scope.

The PICOS approach was employed to define the inclusion criteria: P (patients): All the patients were diagnosed with renal cell carcinoma with venous tumor thrombus; I (intervention): undergone RA-RNTT or L-RNTT; C (comparator): the comparator was O-RNTT; O (outcome): the studies included perioperative outcomes, complications, and oncologic outcomes; S (study type): prospective studies, cohort studies, retrospective studies or randomized controlled trials. The exclusion criteria were as follows: noncomparative studies, editorial comments, letters, case reports, and unpublished studies, and lack of available data for the meta‐analysis.

Data extraction from each qualified literature was independently conducted by two authors. The following data were recorded: (1) First author, publication date, center, propensity score analysis, and country. (2) Age, BMI, sample size, sex, tumor diameter and site, thrombus level (Mayo classification)^16^, surgical approach, and follow-up period. (3) Perioperative outcomes, including hospital stay, operative time, blood loss, transfusion rate, transfusion volume, mortality rate (intraoperative, within 30 days, and total mortality), intraoperative complications, major complications (Clavien grade ≥3), and overall complications (Clavien grade ≥1)^17^. (4) Oncologic outcomes, including local recurrence, overall survival (OS), recurrence-free survival (RFS), cancer-specific survival (CSS), tumor stage, and histopathological results. Disagreements and differences between authors were resolved by reaching a consensus with a third reviewer.

Two reviewers independently assessed the quality of the eligible studies using the risk of bias in nonrandomized studies of interventions (ROBINS-I)^18^, including seven biases due to (1) confounding; (2) selection; (3) exposure classification; (4) deviation from intended exposures; (5) missing data; (6) outcomes measurement; and (7) selection of reported results. Furthermore, a funnel plot was used to examine publication bias.

### Statistical analysis

The Cochrane Collaborative RevMan5.4 software (Cochrane Collaboration, Oxford) was utilized for data analysis. The odds ratio (OR) and weighted mean difference (WMD) were calculated for dichotomous and continuous outcomes, respectively. Moreover, the 95% CI and *I* ^2^ test were used to assess heterogeneity among the included studies^19^. Considering the predictable significance between-trial heterogeneity, the random-effects model was used for all analyses, and *P*<0.05 was considered statistically significant. A sensitivity analysis was conducted for outcomes with significant heterogeneity in order to identify the source of between-study heterogeneity and to assess robustness. However, it was not possible to perform a sensitivity analysis when comparing three or fewer studies.

### Subgroup analysis

Subgroup analyses were performed according to the different surgical approaches for MI-RNTT, RA-RNTT, and L-RNTT.

## Results

### Baseline characteristics

**Figure 1:**
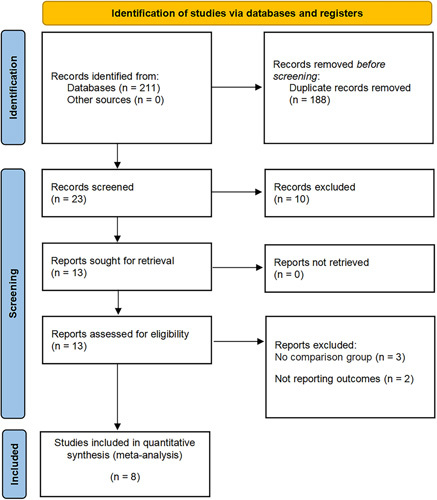
PRISMA (Preferred Reporting Items for Systematic Reviews and Meta-Analyses) flow diagram for the systematic review.

A total of 211 articles were initially searched through electronic search, with 23 remaining after removing duplicates. After reading the titles and abstracts, eight studies involving 563 patients were eligible for inclusion in this meta-analysis (1350 MI-RNTT vs. 1055 O-RNTT) (Fig. 1)^20–27^. All eight studies were retrospective studies, with three studies being multi-institutional^21,24,25^ and two studies being propensity score analyses^20,25^. These studies were performed in different countries, including China, France, the USA, and Germany, and published between 2014 and 2022. The Mayo classification of six studies was level I–II^21–25,27^, one study was level I–III^20^, and one study included part of level 0 cases^26^. The follow-up period ranged from 8 to 79 months. The baseline characteristics of patients included in each literature review are summarized in Tables 1 and 2 (age, BMI, sample size, sex, tumor diameter, site, thrombus level, surgical approach, and follow-up period). Table 3 displays the tumor stage and histopathological results.

**Table 1:** The trials included in the systemic review.

					Patients		Age (years)		Male/Female		BMI (kg/m^2^)		
References	Country	Center	Study design	Propensity score analysis	MI-RNTT	O-RNTT	MI-RNTT	O-RNTT	MI-RNTT	O-RNTT	MI-RNTT	O-RNTT	Surgical approach
Zhang^20^	China	single-center	Retrospective comparative trial	Yes	88	88	60 (10.37)	58 (10.37)	56/32	58/30	24.1 (3.11)	25.4 (4.22)	Laparoscopic versus Open
Vuong^21^	France	multi-institutional	Retrospective comparative trial	No	10	30	73 (7.04)	67 (9.41)	7/3	4/26	27.6 (4.44)	26.8 (3.33)	Robotic versus Open
Liu^22^	China	single-center	Retrospective comparative trial	No	41	46	60.2 (10.66)	56.02 (8.62)	29/12	37/9	23.42 (4.11)	24.11 (3.45)	Laparoscopic versus Open
Rose^23^	USA	single-center	Retrospective comparative trial	No	24	28	63 (8.89)	66 (14.07)	22/2	22/6	31.3 (5.93)	26.8 (5.85)	Robotic versus Open
Beksac^24^	USA	multi-institutional	Retrospective comparative trial	No	11	9	64 (7.25)	65 (13.75)	6/5	8/1	27.7 (6.03)	30.2 (4.18)	Robotic versus Open
Gu^25^	China	multi-institutional	Retrospective comparative trial	Yes	31	31	55.7 (11)	55.3 (12.1)	26/5	24/7	24.8 (2.7)	23.5 (3.9)	Robotic versus Open
Ebbing^26^	Germany	single-center	Retrospective comparative trial	No	31	57	64 (17)	NA	26 (6)	Laparoscopic versus Open			
Xu^27^	China	single-center	Retrospective comparative trial	No	6	32	42.5 (18.6)	57.1 (9.3)	3/3	25/7	22.5 (3.6)	24.3 (3.9)	Laparoscopic versus Open

ASA, American Society of Anesthesiologists; Mean (SD); MI-RNTT, minimally-invasive radical nephrectomy with tumor thrombus; O-RNTT, open radical nephrectomy with tumor thrombus.

**Table 2:** The trials included in the systemic review.

	Tumor site (Lt/Rt)		Tumor diameter (cm)		Thrombus level		ASA Score		Follow-up duration (months)	
References	MI-RNTT	O-RNTT	MI-RNTT	O-RNTT	MI-RNTT	O-RNTT	MI-RNTT	O-RNTT	MI-RNTT	O-RNTT
Zhang^20^	34/53	36/32	6.4 (2.96)	7.2 (2.6)	I: 20; II: 61; III: 7	I: 22; II: 59; III: 7	1: 7; 2: 72; 3: 9; 4: 0	1: 6; 2: 72; 3: 10; 4: 0	31 (19–44)	32 (17–40)
Vuong^21^	4/6	13/17	7 (5.93)	10 (4)	I: 6; II: 4	I: 14; II: 16	1: 0; 2: 3; 3: 7	1: 2; 2: 13; 3: 15	8 (3.8–14.5)	13 (4.3–24.5)
Liu^22^	9/32	10/36	7.95 (2.16)	10.10 (4.17)	I: 26; II: 15	I: 14; II: 32	1: 2; 2: 36; 3: 3	1: 2; 2: 40; 3: 4	Mean: 2	
Rose^23^	4/20	7/21	NA		I: 2; II: 22	I: 6; II: 22	2: 4; 3: 14; 4: 0	2: 3; 3: 22; 4: 3	24 (7–41)	79 (21–112)
Beksac^24^	3/8	1/8	8.4 (2.68)	10 (3.13)	II		NA		NA	
Gu^25^	6/5	5/6	7.3 (3.0)	8.4 (2.3)	I: 13; II: 18	I: 10; II: 21	1+2: 25	1+2: 23	Mean: 27	Mean: 44.8
Ebbing^26^	NA		6.0 (2.0)	8.5 (3.0)	0-I		NA		23.5 (1–116)	
Xu^27^	1/5	9/23	7.0 (0.8)	9.4 (2.4)	I: 4; II: 2	I: 13; II: 19	NA		Mean: 18.2	

Mean (SD); MI-RNTT, minimally-invasive radical nephrectomy with tumor thrombus; O-RNTT, open radical nephrectomy with tumor thrombus.

**Table 3:** Oncologic outcomes.

	Tumor stage		Tumor pathology	
References	MI-RNTT	O-RNTT	MI-RNTT	O-RNTT
Zhang^20^	pT3a:15; pT3b:39; pT3c:36; pT4:4	pT3a:11; pT3b:34; pT3c:39; pT4:4	Clear cell: 78; Papillary: 9; Chromophobe: 1; Others: 0	Clear cell: 73; Papillary: 11; Chromophobe: 0; Others: 4
Vuong^21^	pT3b:10; pT4:0; N+: 5; M+: 2	pT3b:28; pT4:2; N+: 11; M+: 10	Clear cell: 8; Papillary: 2	Clear cell: 8; Papillary: 2
Liu^22^	cN0:18; cN1:23; cM0:32; cM1:9	cN0:16; cN1:30; cM0:33; cM1:13	Clear cell: 37; Others: 4	Clear cell: 37; Others: 9
Rose^23^	M1:5	M1:8	Clear cell: 20; Others: 4	Clear cell: 25; Others: 3
Beksac^24^	pT3a:8; pT3b:2; pT4:1; pN0:4; pN1:2; pNx:5; pM0:0; pM1:1; pMx:10	pT3a:5; pT3b:4; pT4:0; pN0:5; pN1:1; pNx:3; pM0:1; pM1:0; pMx:8	Clear cell: 8; Papillary: 0; Chromophobe: 2; Leiomyosarcoma: 1	Clear cell: 8; Papillary: 0; Chromophobe: 0; Leiomyosarcoma: 1
Gu^25^	T3b:31; T4:0	T3b:30; T4:1	Clear cell: 26; Papillary: 3; Chromophobe: 0; Others: 2	Clear cell: 26; Papillary: 2; Chromophobe: 1; Others: 2
Ebbing^26^	T3a		NA	
Xu^27^	NA		NA	

MI-RNTT, minimally-invasive radical nephrectomy with tumor thrombus; O-RNTT, open radical nephrectomy with tumor thrombus.

There was no statistically significant difference in age (*P*=0.26), side of the tumor (*P*=0.74), and BMI (*P*=0.75) between the two groups. Nevertheless, the tumor diameter was smaller in the MI-RNTT group compared to the O-RNTT group (*P*<0.0001) (Table S1, Supplemental Digital Content 3, http://links.lww.com/JS9/A790).

### Assessment of quality

Six studies exhibited a moderate risk of bias^21–24,26,27^, whereas two studies had a low risk of bias^20,25^, and all studies conducted a comparative analysis (Table S2, Supplemental Digital Content 4, http://links.lww.com/JS9/A791).

### Outcome analysis

#### Perioperative effectiveness

**Figure 2:**
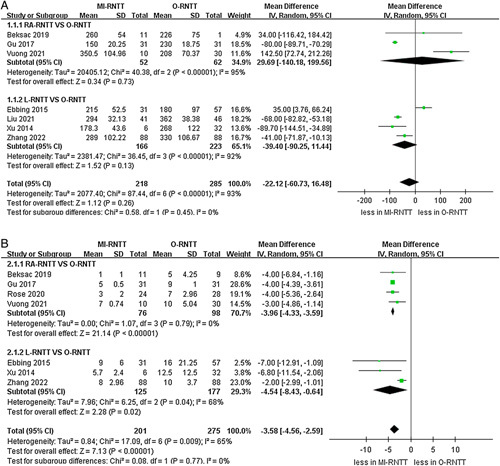
Forest plots of perioperative outcomes for minimally-invasive radical nephrectomy with tumor thrombus versus open radical nephrectomy with tumor thrombus. (A) operative time and (B) length of hospital stay.

A meta-analysis of the operative time revealed no statistically significant difference between the MI-RNTT and O-RNTT groups (seven studies pooled; *P*=0.26)^20–22,24–27^. In addition, subgroup analysis demonstrated that there were no statistically significant differences between RA-RNTT and L-RNTT in terms of operative time compared to O-RNTT (*P*=0.73; *P*=0.13). Notably, the MI-RNTT group was associated with a shorter length of hospital stay than the O-RNTT group (WMD -3.58 days, 95% CI: −4.56 to −2.59; *P*<0.00001); seven studies^20,21,23–27^, as well as the subgroup analysis, illustrated that length of stay for RA-RNTT and L-RNTT were shorter than for O-RNTT (RA-RNTT: WMD -3.96 days, 95% CI: −4.33 to −3.59; *P*<0.00001; L-RNTT: WMD -4.54 days, 95% CI: −8.43 to −0.64; *P*=0.02) (Fig. 2).

**Figure 3:**
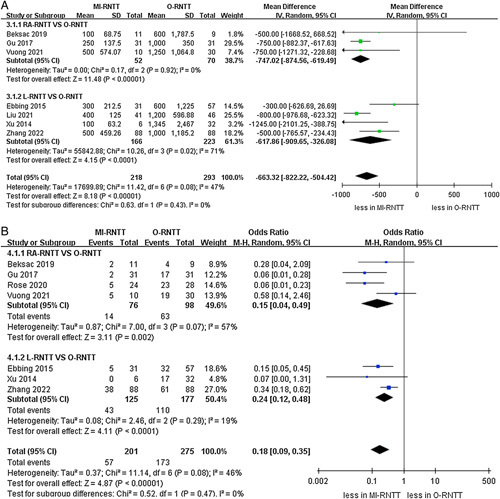
Forest plots of perioperative outcomes for minimally-invasive radical nephrectomy with tumor thrombus versus open radical nephrectomy with tumor thrombus. (A) blood loss and (B) transfusion rates.

**Figure 4:**
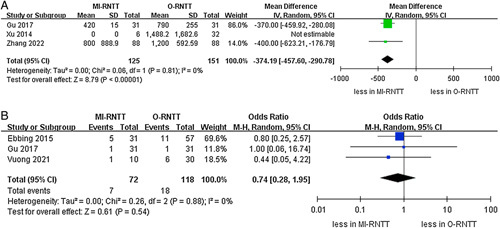
Forest plots of perioperative outcomes and complications for minimally-invasive radical nephrectomy with tumor thrombus versus open radical nephrectomy with tumor thrombus. (A) transfusion volumes and (B) intraoperative complications.

The pooled results demonstrated that the MI-RNTT group had lower volumes of estimated blood loss than the O-RNTT group (WMD -663.32 ml, 95% CI: −822.22 to −504.42; *P*<0.00001)^20–22,24–27^. Furthermore, the subgroup analysis revealed that both the RA-RNTT and L-RNTT groups were correlated with lower amounts of blood loss compared to the O-RNTT group (RA-RNTT: WMD −747.02 ml, 95% CI: −847.56 to −619.49; *P*<0.00001; L-RNTT: WMD -617.86 ml, 95% CI: −909.65 to −326.08; *P*<0.00001). Meanwhile, MI-RNTT was associated with fewer transfusion rates compared to O-RNTT (OR 0.18, 95% CI: 0.09–0.35; *P*<0.00001) in seven studies^20,21,23–27^. Similar results were found in the RA-RNTT and L-RNTT subgroups (RA-RNTT: OR 0.15, 95% CI: 0.04–0.49; *P*=0.002; L-RNTT: OR 0.24, 95% CI: 0.12–0.48; *P*<0.0001) (Fig. 3). Lastly, the pooled results of three studies demonstrated that MI-RNTT was associated with lower transfusion volumes compared to O-RNTT (WMD -374.19 ml, 95% CI: −457.60 to −290.78; *P*<0.00001) (Fig. 4)^20,25,27^.

#### Complications

**Figure 5:**
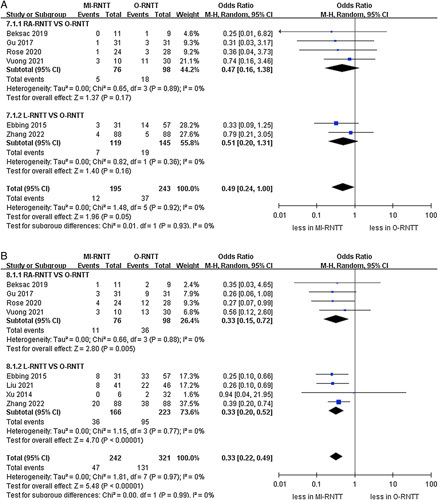
Forest plots of complication for minimally-invasive radical nephrectomy with tumor thrombus versus open radical nephrectomy with tumor thrombus. (A) major complications and (B) overall complications.

The cumulative analysis revealed showed no significant difference in the prevalence of intraoperative complications (three studies; *P*=0.54) (Fig. 4)^21,25,26^. However, the pooled results established that the MI-RNTT group was linked to fewer major complications than the O-RNTT group (OR 0.49, 95% CI: 0.24–1.00; *P*=0.05, six studies)^21–26^, while subgroup analysis reported that there was no statistically significant difference between RA-RNTT and L-RNTT compared to O-RNTT regarding major complications (*P*=0.17; *P*=0.16). The meta‐analysis included eight studies that reported the overall complications^20–27^. Overall complication rates were 19.4% (47 out of 242 cases) in the MI-RNTT group and 40.8% (131 of 321 cases) in the O-RNTT group. Similarly, the pooled results demonstrated that MI-RNTT was associated with fewer overall complications than O-RNTT (OR 0.33, 95% CI: 0.22–0.49; *P*<0.00001) (Fig. 5).

#### Oncologic outcomes

**Figure 6:**
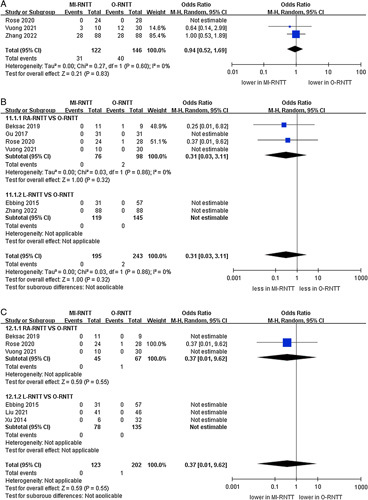
Forest plots of mortality rates for minimally-invasive radical nephrectomy with tumor thrombus versus open radical nephrectomy with tumor thrombus. (A) total mortality rate, (B) 30-day mortality rate, and (C) intraoperative mortality rate.

No significant differences were noted regarding total and intraoperative mortality rates between the MI-RNTT and O-RNTT groups (three studies; *P*=0.83, six studies; *P*=0.55)^21,23,2621–24,26,27^. Similarly, no statistically significant difference in 30-day mortality rates was observed between the two groups (six studies; *P*=0.32)^20,21,23–26^. Moreover, the subgroup analysis also demonstrated that there were no statistically significant differences in 30-day mortality rates between the MI-RNTT and L-RNTT subgroups compared to the O-RNTT group (Fig. 6).

**Figure 7:**
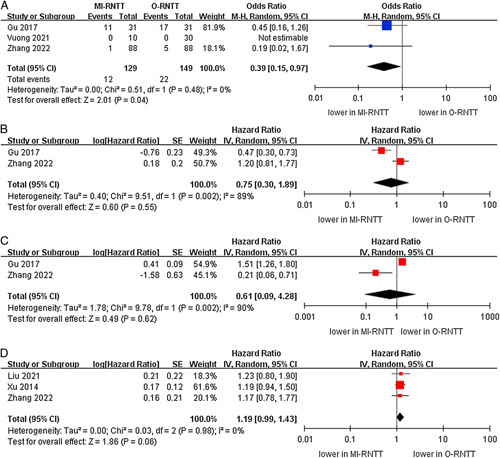
Forest plots of oncologic outcomes for minimally-invasive radical nephrectomy with tumor thrombus versus open radical nephrectomy with tumor thrombus. (A) local recurrence, (B) overall survival, (C) recurrence-free survival, and (D) cancer-specific survival.

In terms of local recurrence, recurrence was reported in three studies^20,21,25^. MI-RNTT was associated with lower recurrence compared to O-RNTT (OR 0.39, 95% CI: 0.15–0.97; *P*=0.04). More importantly, the cumulative analysis revealed no significant difference in the prevalence of CSS between the two groups (three studies; *P*=0.06)^20,22,27^. Likewise, there was also no significant difference in the OS and RFS between the MI-RNTT and O-RNTT groups (two studies; *P*=0.55, two studies; *P*=0.62) (Fig. 7)^20,25^.

### Heterogeneity

Most of the results showed low to moderate heterogeneity. Although the included studies were of intermediate and high quality, high heterogeneity was observed in four outcomes (operative time, *I* ^2^=93%; length of hospital stay, *I* ^2^=65%; OS, *I* ^2^=89%; RFS, *I* ^2^=90%).

### Sensitivity analysis

Herein, owing to the evident heterogeneity in operative time and length of stay, sensitivity analysis was conducted to quantify the source of heterogeneity and to evaluate the robustness of the results. Finally, no substantial change in heterogeneity was found between the two outcomes, indicating that the source of heterogeneity in operative time and length of hospital stay was steady.

### Publication bias

Publication bias was examined by calculating the indexes of operative time, length of stay, blood loss, and overall complications. The findings presented a lack of symmetry between studies, suggesting the presence of publication bias (Fig. S1).

## Discussion

The present study provided crucial findings regarding perioperative complications and oncologic outcomes that warrant further discussion.

Some studies have reported that a robotic-assisted procedure was associated with a longer operative time compared to an open procedure, most likely attributable to the time required for the robot arm to dock^28^. Interestingly, no statistically significant difference in operative time was observed between the RA-RNTT and O-RNTT groups. All robotic and laparoscopic interventions were performed by operators with abundant experience in minimally-invasive surgery, and this may partly explain this outcome. Furthermore, owing to the limited space in minimally-invasive surgery, tumor diameter, laterality, and thrombus level have become important parameters to consider. Besides, no statistically significant difference in laterality was observed between the MI-RNTT and the O-RNTT groups; however, the tumor size was smaller in the MI-RNTT group. Thus, it is essential to exercise caution when interpreting results such as the operative time between the MI-RNTT and O-RNTT. In terms of length of hospital stay, the meta-analysis illustrated that the length of stay in the RA-RNTT and L-RNTT subgroups was lower than in the O-RNTT. Robotic and laparoscopic surgeries can result in less trauma, faster intestinal function recovery, and fewer bed rest-associated complications, thus potentially leading to a shorter hospital stay^29,30^. Moreover, rapid developments in anesthesiology and perioperative care management could also lead to a reduction in the length of stay. Two studies reported that there were no significant differences in postoperative intensive care monitoring duration between the MI-RNTT and O-RNTT groups; however, the duration was shorter in the MI-RNTT group compared to the O-RNTT group^21,26^. Therefore, more high-quality studies are necessary to validate this outcome.

One of the major advantages of minimally-invasive surgery appears to be reduced blood loss. The robotic platform is capable of delivering meticulous hemostasis owing to its articulated instruments, whilst robotic and laparoscopic vision imaging systems can potentially expand the surgical field during dissection. Furthermore, the pneumoperitoneum established by the minimally-invasive surgery serves to mitigate venous hemorrhages during dissection^31^. The aforementioned reasons might explain the lower blood loss volume in the MI-RNTT group compared to the O-RNTT group. For the first time, Zhang *et al*.^20^ conducted a study to evaluate the perioperative and oncological outcomes of robotic and laparoscopic radical nephrectomy with venous thrombectomy in 110 patients and determined that RA-RNTT was associated with a lower blood loss volume than L-RNTT. However, given that reports on this topic are limited, a reliable conclusion cannot be reached, and thus further studies are required to assess the outcomes in the two groups. Similarly, MI-RNTT was associated with fewer transfusion rates and transfusion volumes compared to O-RNTT.

In the present study, the pooled results revealed that the MI-RNTT group was associated with fewer overall and major complications than the O-RNTT group. The incision area and anatomical scope are smaller in minimally-invasive surgery, which results in decreased trauma and, subsequently, fewer complications^32^. It is worth emphasizing that thromboembolism is a fatal complication. One included study reported that the rate of thromboembolism was 7% in the open group and 8% in the robotic group^23^. Meanwhile, Shah *et al*.^33^ conducted a retrospective study and reported that the rate of thromboembolism in patients who had undergone O-RNTT was ~30% after a follow-up duration of 12 months. While there were no fatalities reported due to thromboembolism during the operation in the included studies, caution should be taken during the manipulation of thrombectomy. Furthermore, one study demonstrated that the learning stage was one of the independent predictive factors of early complications. Other influencing factors included the length of the thrombus, thrombus level, and inferior vena cava management technique^34^. Therefore, long-term follow-up studies should be conducted to further validate the results regarding complications.

Shao *et al*.^10^ also suggested that thrombectomy should be conducted with care due to the reported mortality rate of 4–10%. Interestingly, no patients died as a result of thromboembolism within 30 days in the included studies. Herein, no significant differences were noted regarding intraoperative, 30-day, and total mortality rates between the two groups. This critical finding demonstrates that experienced laparoscopic and robotic surgeons could adequately ensure patient safety. Furthermore, Rose *et al*.^23^ described that the use of prophylactic anticoagulation can effectively contribute to perioperative care management. Consequently, they adopted preoperative anticoagulation for all patients irrespective of active bleeding or not, and postoperative prophylactic anticoagulation was administered for 30 days. Nonetheless, further high-quality studies should be conducted to verify its curative effect.

Oncologic outcomes are key indicators of surgical quality. MI-RNTT was associated with lower recurrence compared to O-RNTT. Nevertheless, some critical issues must be taken into account when comparing the oncologic outcomes between the two groups. To begin, three included studies^20,21,23^ reported that some patients received adjuvant therapy, and postoperative adjuvant targeted drugs may potentially prolong the time for local recurrence. Therefore, this result should be interpreted with caution. Secondly, because of the relatively small number of local recurrent events in both groups, it is likely that the surgery may not play a decisive role in determining recurrence. Although there was no statistically significant difference in CSS, OS, and RFS between the two groups, the short follow-up period in most of the included studies and the insufficient number of studies hindered the generation of reliable conclusions.

Most of the included studies have predominantly described cases with level I–II thrombus, while only one study included a few cases with level III thrombus. In 2015, Shao *et al*.^10^ performed L-RNTT for level IV thrombus for the first time and demonstrated that L-RNTT could be a safe and effective alternative to open surgery for level IV thrombus. Furthermore, Chopra *et al*.^35^ conducted a study to evaluate the efficacy and safety of RA-RNTT for level III thrombus and revealed that RA-RNTT is a viable approach for level III thrombus. However, there is no comparative study between MI-RNTT and O-RNTT for level III-IV thrombus, thus warranting further exploration into the efficacy of minimally-invasive surgical procedures in this situation. Concerning nephrectomy, the included studies applied different surgical methods, such as the transperitoneal or retroperitoneal approach. The latter offers certain benefits, such as the ability to ligate the renal artery more easily, thus limiting hemorrhage during renal tumor excision. Moreover, it reduces the likelihood of interference with the intestine, thus minimizing the risk of complications^36^. Nevertheless, compared with the transperitoneal approach, the retroperitoneal approach also has drawbacks, such as limited space. Therefore, further research with higher-quality evidence is warranted to determine the most suitable surgical approach (transperitoneal or retroperitoneal) for MI-RNTT.

This study was conducted in accordance with the rigorous guidelines of PRISMA^13^ (Supplemental Digital Content 1, http://links.lww.com/JS9/A788); nonetheless, there were still some limitations in the analysis. Firstly, all of the studies were retrospective with intermediate to high quality, and none of the studies were randomized controlled trials; therefore, the studies must have undoubtedly been influenced by inherent selection biases. Secondly, the included studies in this review contained a heterogeneous level of thrombus (I–III). It was not possible to conduct subgroup analyses based on the thrombus level given the insufficient literature and the nature of the included literature, which may have further contributed to heterogeneity. Thirdly, the included studies employed different surgical approaches (transperitoneal or retroperitoneal); again, the limited literature precluded us from performing subgroup analysis, potentially leading to subtle differences. Fourth, regarding oncologic outcomes such as the CSS, OS, and RFS, only two or three studies were included, resulting in less reliable results. Lastly, long-term follow-up oncologic outcomes could not be assessed from the included studies, thus preventing comparisons between the two groups in terms of oncological outcomes.

## Conclusions

The current outcomes suggest that MI-RNTT is a safe and effective procedure that yields superior outcomes to O-RNTT in the treatment of renal cell carcinoma with venous tumor thrombus, including shorter length of stay, lower blood loss volumes, and fewer complications. Moreover, minimally-invasive surgery has demonstrated comparable mortality rates and oncological outcomes to open surgery. However, more comprehensive and rigorous research is necessitated to further corroborate the findings of this study, which should include a larger sample size and comprehensive data from high-volume medical centers.

## Ethical approval

Not applicable.

## Sources of funding

This study was supported by the National Natural Science Foundation of China (No. 82160146); Cuiying Scientific and Technological Innovation Program of Lanzhou University Second Hospital (Grant numbers CY2021-MS-A12 and CY2020- MS08); Natural Science Foundation of Gansu Province of China (Grant numbers 21JR1RA151); Second Hospital of Lanzhou University ‘Cuiying Science and Technology Innovation’ project (CY2021-QN-A20).

## Conflicts of interest disclosure

The authors declare that they have no conflicts of interest.

## Author contribution

K.-P.L. and S.-Y.C.: conceived and designed the experiments; K.-P.L., S.-Y.C., X.-R.L., and L.Y.: analyzed the data; K.-P.L., S.-Y.C., and C.-Y.W.: contributed reagents/materials/analysis; K.-P.L., S.-Y.C., C.-Y.W., X.-R.L., and L.Y.: wrote the manuscript. All authors have read and approved the final manuscript.

## Research registration unique identifying number (UIN)

1. Name of the registry: PROSPERO database.
2. Unique identifying number or registration ID: CRD42022381118.
3. Hyperlink to your specific registration (must be publicly accessible and will be checked): https://www.crd.york.ac.uk/prospero/display_record.php?RecordID=381118.

## Guarantor

Li Yang.

## Data availability statement

All data generated and analyzed during this study are included in this published article. The data presented in the article may be requested by consulting the correspondence author.

## Supplementary Material

SUPPLEMENTARY MATERIAL
