# Supplementary material for: Perioperative and oncologic outcomes of minimally-invasive surgery for renal cell carcinoma with venous tumor thrombus: a systematic review and meta-analysis of comparative trials
Source: Int J Surg. 2023 Jul 31;109(9):2762–73. doi: 10.1097/JS9.0000000000000405 (PMC10498880; doi:10.1097/JS9.0000000000000405)
Supplement: SUPPLEMENTARY MATERIAL [file js9-109-2762-s003.docx]

| **Table S1 Comparison of baseline patient** | | | |  |
| --- | --- | --- | --- | --- |
| Baseline characteristic | MI-RNTT VS O-RNTT | Heterogeneity I^2^ (%) | *p* value |  |
| Age WMD (95% CI) | 1.43(-1.46 to 4.32) | 44 | 0.33 |  |
| Left side OR (95% CI) | 0.93(0.61 to 1.42) | 0 | 0.74 |  |
| BMI WMD (95% CI) | 0.24(-1.27 to 1.76) | 70 | 0.75 |  |
| Tumor diameter WMD (95% CI) | -1.78(-2.44 to -1.12) | 41 | < 0.00001 |  |
| MI-RNTT: Minimally-invasive radical nephrectomy with tumor thrombus; O-RNTT: Open radical nephrectomy with tumor thrombus; WMD = weighted mean difference; CI = confidence interval; OR = odds ratio | | | |  |
|  |  |  |  |  |
