# Supplementary material for: Perioperative and oncologic outcomes of minimally-invasive surgery for renal cell carcinoma with venous tumor thrombus: a systematic review and meta-analysis of comparative trials
Source: Int J Surg. 2023 Jul 31;109(9):2762–73. doi: 10.1097/JS9.0000000000000405 (PMC10498880; doi:10.1097/JS9.0000000000000405)
Supplement: SUPPLEMENTARY MATERIAL [file js9-109-2762-s004.docx]

| **Table S2 The risk of bias(Non-RCTs)-ROBINS-I** | | | | | | | | |  |
| --- | --- | --- | --- | --- | --- | --- | --- | --- | --- |
| Bias domain | Zhang | Vuong | Liu | Rose | Beksac | Gu | Ebbing | Xu |  |
|  |  |  |  |  |  |  |  |  |  |
| Bias due to confounding | Moderate | Moderate | Moderate | Moderate | Moderate | Moderate | Moderate | Moderate |  |
|  |  |  |  |  |  |  |  |  |  |
| Bias in selection of participants into the study | Low | Low | Low | Low | Low | Low | Low | Low |  |
|  |  |  |  |  |  |  |  |  |  |
| Bias in classification of interventions | Low | Low | Low | Low | Low | Low | Low | Low |  |
|  |  |  |  |  |  |  |  |  |  |
| Bias due to deviations from intended interventions | Low | Low | Moderate | Moderate | Low | Moderate | Moderate | Moderate |  |
|  |  |  |  |  |  |  |  |  |  |
| Bias due to missing data | Moderate | Moderate | Low | Low | Moderate | Low | Low | Low |  |
|  |  |  |  |  |  |  |  |  |  |
| Bias in measurement of outcomes | Low | Low | Moderate | Low | Moderate | Low | Low | Low |  |
|  |  |  |  |  |  |  |  |  |  |
| Bias in selection of the reported result | Low | Moderate | Moderate | Moderate | Moderate | Low | Moderate | Moderate |  |
|  |  |  |  |  |  |  |  |  |  |
| Overall bias | Low | Moderate | Moderate | Moderate | Moderate | Low | Moderate | Moderate |  |
|  |  |  |  |  |  |  |  |  |  |
